# Endovascular recanalization of extensively-thrombosed cerebral venous sinuses in early pregnancy: A case report

**DOI:** 10.1097/MD.0000000000030266

**Published:** 2022-09-09

**Authors:** Kun Zhang, Tian-Xiao Li, Bu-Lang Gao, Liang-Fu Zhu, Zi-Liang Wang

**Affiliations:** a Cerebrovascular Center, Henan Provincial People’s Hospital, Zhengzhou University, Zhengzhou, Henan Province, China.

**Keywords:** aspiration, cerebral hemorrhage, cerebral venous sinus thrombosis, endovascular treatment, Navien catheter

## Abstract

The incidence of pregnancy-related cerebral venous sinus thrombosis (CVST) is rare, and cerebral hemorrhage caused by CVST in the early pregnancy period is even rarer. Only 3 cases of cerebral hemorrhage caused by CVST had been reported in the early pregnancy period in the literature. In this study, we reported successful endovascular treatment of such a case.

**Patient concerns:**

A 27-year-old woman presented with rapidly progressive neurologic decline in her second pregnancy for 8 weeks. She was afebrile and completely conscious, without neurological deficits. She did not have any previous history of venous thrombosis, hematologic, or autoimmune diseases.

**Diagnosis:**

Urgent brain computed tomography demonstrated parietal-occipital hemorrhage surrounded by a large hypodense area and full brain swelling. Magnetic resonance venography showed complete occlusion of the right sigmoid sinus, transverse sinus, and two-thirds of the superior sagittal sinus. Transvaginal sonography demonstrated early intrauterine pregnancy, with the size of gestation sac being 6 × 7 × 6 mm and the fetal heart not being detected. CVST-related cerebral hemorrhage was confirmed based on the clinical and imaging data.

**Interventions:**

The CVST in this pregnant woman was treated endovascularly with a 6 Fr Navien catheter for aspiration, thrombolysis, and anticoagulation.

**Outcomes:**

Ten days after treatment, the cerebral hemorrhage had gradually been absorbed. Follow-up angiography performed 2 weeks later demonstrated complete recanalization of her cortical veins and sinuses. Two months later, the patient was completely recovered without cognitive or neurological dysfunction.

**Lessons:**

Pregnancy-related CVST can be successfully treated with a combined endovascular approach of aspiration, thrombolysis, and anticoagulation to complete recovery.

## 1. Introduction

Thrombosis within the cerebral venous sinus thrombosis (CVST) was first reported as a cause of death in the early 19th century and now takes up about 0.5% to 1.0% among all strokes.^[1,2]^ Studies reporting the prevalence of pregnancy-related CVST in Asian countries are rare, and some studies have demonstrated that the prevalence of pregnancy-related CVST was 6.3%. This illness has a mortality rate ranging from 5% to 30%.^[3]^ CVST rarely causes cerebral hemorrhage, and CVST-related cerebral hemorrhage in the early pregnancy period is extraordinarily rare.^[4,5]^ Only 3 cases of CVST-related cerebral hemorrhage in the early pregnancy period had been reported in the literature.^[4–6]^ In this study, we reported a 27-year-old woman who developed CVST-related cerebral hemorrhage in her eighth week of pregnancy and was successfully treated with a 6 Fr Navien for aspiration, thrombolysis, and anticoagulation. To the best of our knowledge, this was the first case who had been treated with a combined endovascular approach to complete recovery.

The Navien^TM^ Intracranial Support Catheter (Covidien Vascular Therapies, Mansfield, MA) is a novel intracranial catheter which shows self-anchoring at curved sinuses. We demonstrated a novel approach utilizing a 6 Fr Navien catheter through transvenous access by the femoral vein to navigate an aspiration catheter, and use of the relatively big catheter leads to better outcomes for percutaneous catheter aspiration of sinus thrombi. This was the first case of CVST-associated quickly aggravated malignant brain edema and nearly-fatal herniation which was successfully treated with a combined endovascular approach of local urokinase thrombolysis, systemic heparin anticoagulation, and Navien catheter aspiration.

## 2. Case report

**Figure 1.**
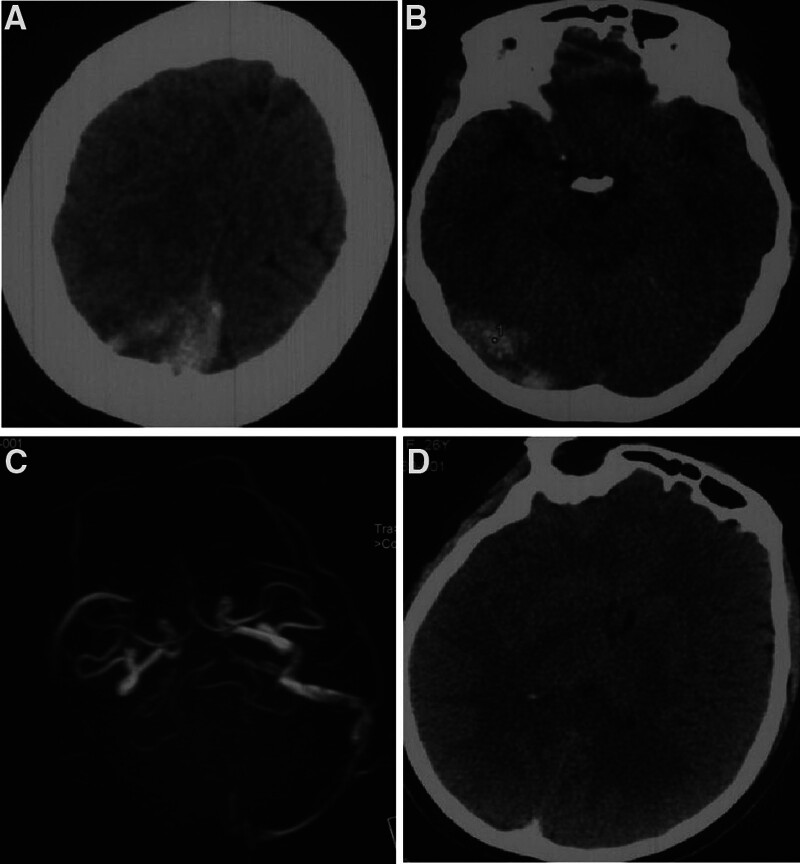
A 27-year-old woman in her early pregnancy had cerebral hemorrhage. (A and B) Non-contrast head computed tomography (CT) demonstrated occipital hemorrhage surrounded by a large hypodense area. (C) Magnetic resonance imaging (MRI) revealed absence of continuous flow within the superior sagittal sinus (SSS), the right transverse sinus (TS), and the right ligmoid sinus. (D) CT scan showed diffuse brain swelling with compressed ventricles.

**Figure 2.**
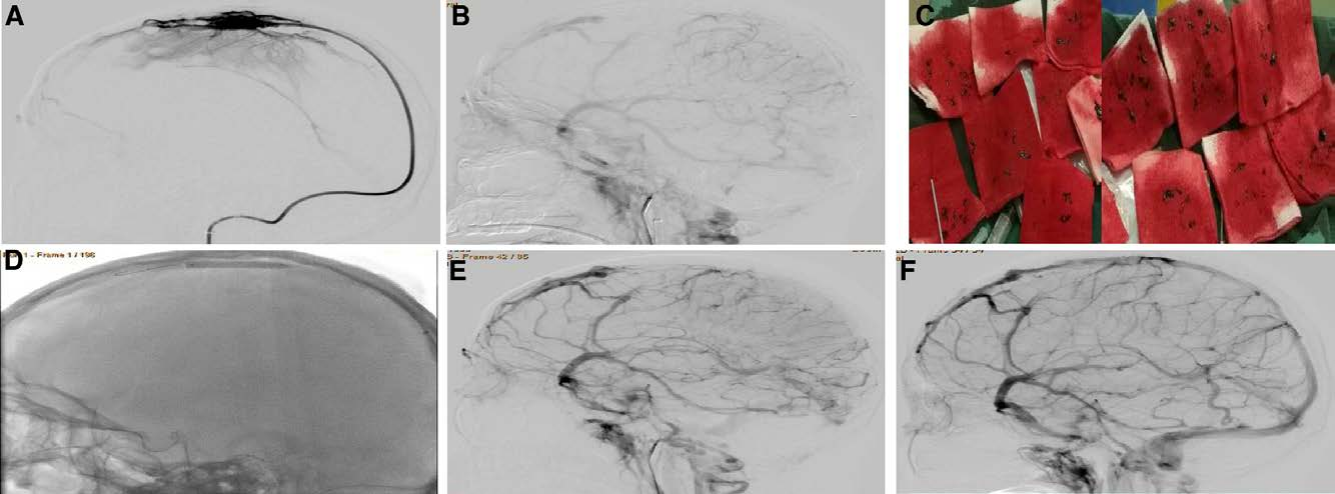
Endovascular treatment was performed for the patient with thrombosed cerebral venous sinuses complicated with cerebral hemorrhage. (A) Digital subtraction angiography with a 6 Fr Navien catheter in the superior sagittal sinus (SSS) showed poor anterograde flow with multiple filling defects in the SSS, transverse sinus (TS), and sigmoid sinus (SS). (B) Lateral internal carotid artery injection angiography demonstrated no contrast within the superficial or deep venous sinus system. (C) Many clots were removed from the 6 Fr Navien catheter. (D) An ultra-soft 4.0 × 30 mm balloon was then sent to the anterior portion of the thrombosed SSS for treatment. (E) Right internal carotid artery angiography revealed improved intraluminal flow with partial obstruction of the SSS (arrows). (F) Right angiography 2 weeks after the intervention showed complete patency of the previously occluded SSS and cortical veins with normal arteriovenous transit time.

This case study was approved by the Ethics Committee of Henan Provincial People’s Hospital, with informed consent obtained from the patient. A 27-year-old female patient presented with rapidly progressive neurologic decline caused by malignant edema of the brain associated with extensive thrombosis within the superior sagittal sinus (SSS) and cortical veins in spite of intravenous heparin administration. She was having her second pregnancy for 8 weeks. She did not have any previous history of venous thrombosis, hematologic, or autoimmune diseases. She was afebrile and completely conscious, without neurological deficits. Laboratory test revealed normal blood counts and normal profiles in renal, hepatic, and coagulation function. Urgent brain computed tomography (CT) demonstrated parietal-occipital hemorrhage surrounded by a large hypodense area (Fig. 1A and B) and full brain swelling (Fig. 1C). Magnetic resonance venography (MRV) showed complete occlusion of the right sigmoid, transverse, and two-thirds of the SSS (Fig. 1D). Transvaginal sonography demonstrated early intrauterine pregnancy, with the size of gestation sac being 6 × 7 × 6 mm and the fetal heart not being detected. CVST-related cerebral hemorrhage was confirmed based on the clinical and imaging data. Lumbar puncture showed an opening pressure 47 cm H_2_O, 70 red blood cells/mL, 0 white blood cells/mL, glucose 61 mg/dL, and protein 35 mg/dL with negative gram staining. To prevent and control intracranial hypertension, mannitol and furosemidum were administered immediately, and low-molecular-weight heparin in the dose of 9600 units/day for 3 days was started 1 day after disease onset. But 2 days after hospitalization, the patient’s consciousness was aggravated, and herniation signs were shown together with right pupil dilation. Emergent CT scan revealed full brain swelling (Fig. 1D). Given the progressive thrombotic condition and clinical aggravation despite medication, the patient was treated immediately with endovascular approaches after informed consent to the treatment. Initial diagnostic angiography demonstrated near complete occlusion of the superior sagittal, right transverse and sigmoid sinuses, with vascular engorgement of the superficial veins in bilateral hemispheres and occlusion of multiple cortical veins, leading to severe delay in the arteriovenous transit time (Fig. 2A and B). No signs of arteriovenous malformation and dural arteriovenous fistula were present.

An 80 cm long 6 Fr sheath was advanced into the right internal jugular vein. Next, a 6 Fr Navien catheter was sent to the anterior part of the thrombosed SSS over a coaxially inserted Echelon-10 microcatheter (EV3) and Traxcess 14 micro guidewire. Clot aspiration was performed through the 6 Fr Navien catheter using a 20 mL syringe, and large thrombotic fragments were aspirated and removed from the Navien catheter (Fig. 2C). An ultra-soft SV 4.0 × 30 mm balloon was then sent to the anterior part of the SSS through the Navien catheter, and the balloon was inflated and pulled from the anterior to the posterior portion close to the 6 Fr Navien (Fig. 2D). Then, the balloon was deflated, and aspiration was started through the 20 mL syringe connected to the Navien catheter. Once again, the balloon was sent to the anterior portion of the SSS and was pulled back to the Navien catheter for aspiration through the catheter. The same action was repeated several times, with a lot of large clot fragments being aspirated and removed from the Navien catheter (Fig. 2C). Antegrade flow was established in the SSS (Fig. 2E), the cerebral angiographic cycle was reduced to 18 seconds, but residual wall adherent thrombus remained in the SSS. After the Echelon-10 microcatheter was advanced into the residual wall adherent thrombus, the micro guidewire was pushed back and forth to cut the thrombus, and the microcatheter was navigated to the distal SSS for infusion of 500,000 Unit urokinase (10,000 units/min) for thrombolysis. No significant improvement in flow was shown in angiography; the Echlon-10 microcatheter was then kept in the venous system, and the patient was returned to the ward with the score of the National Institute of Health Stroke Scale being 1.

Over the course of 10 days of hospitalization, the patient had full-dose intravenous normal heparin (12,500 units/day) through the Echlon-10 microcatheter kept in the sinus, and local urokinase thrombolysis (1 million units/day) was also continuously infused into the SSS for 10 days through the microcatheter. Infusion of both normal heparin and urokinase was discontinued after angiography confirmed patency of the SSS. Normal heparin (3 mg/day) was used, and oral warfarin was administered 10 days after the endovascular operation to maintain the international normalized ratio value of coagulation function between 2.0 and 3.0 when a repeated CT scan demonstrated that the cerebral hemorrhage had gradually been absorbed. Abortion was performed 20 days after disease onset. Follow-up angiography performed 2 weeks after the intervention demonstrated complete recanalization of her cortical veins and sinuses (Fig. 2F). Two months later, the patient was completely recovered without cognitive or neurological dysfunction (modified Rankin Scale score of 0).

## 3. Discussion

CVST is an infrequent but severe neurological emergency, and pregnancy induces specific pathophysiological changes, including anemia of iron deficiency, coagulation alterations, changes in the function and levels of prothrombotic and antithrombotic proteins, and hemorrhage during labor and delivery.^[7]^ The greatest risk for CVST in pregnancy mainly occurred in the third trimester,^[5]^ and patients may die of substantial cerebral venous infarction accompanied by severe cerebral edema. Only a few patients with CVST have been reported in the early pregnancy period, and CVST-related cerebral hemorrhage in the early pregnancy is extraordinarily rare.^[3–6,8]^

Imaging features are extremely important in correct diagnosis of CVST.^[9–13]^ Cerebral infarction, edema, and hemorrhage can be found on CT imaging, and a hyperdense lesion of thrombosis can be detected occasionally in the venous sinus.^[9,10,12]^ However, CT scanning is detrimental to pregnant women because of the radiation. Magnetic resonance imaging (MRI) is able to distinguish CVST from tumors, and MRV can detect sinus occlusion.^[10–13]^ Although cerebral angiography is invasive with radiation, this approach can detect sinus thrombosis. Other vascular abnormalities, including aneurysms, arteriovenous fistulas, and arteriovenous malformation, can be excluded by cerebral angiography. MRI and MRV are non-invasive without radiation, and therefore, MRI combined with MRV is the best approach for detecting possible CVST in pregnant females.

We used the combination of Navien aspiration, thrombolysis, and anticoagulation therapy for this case for several reasons. Compared to other microcatheters, the Navien catheters have the minimal outer diameter but greatest inner diameter,^[14]^ with strengths of self-anchoring at curved vessels and maintaining a circular shape without altering to an oval shape in the procedure. These characteristics and the relatively big inner diameter can ensure removal of large clot fragments at aspiration and maintain patent catheter lumen in tortuous vessels for good manipulation and stability of the catheter.^[15]^ Percutaneous intra-sinus thrombolysis was performed using urokinase perfusion via the microcatheter placed into the venous sinus thrombus, which plays a very important role in complete recanalization of the thrombosed cerebral venous sinuses.^[16]^ The decision to treat the patient with urokinase was based on physicians’ preference and availability of thrombolytic drugs.^[17]^ Balloon angioplasty may be used with or without stent deployment in certain situations, even though there is scant literature in support of this method.^[18]^ Nonetheless, we used this approach for complete recanalization of the thrombosed cerebral venous sinuses in this case.

While systemic anticoagulation is mainly to prevent thrombosis propagation, endovascular management can immediately revascularize and restore normal venous flow.^[19]^ We had used the Navien catheters of various sizes specifically designed for application in the intracranial vasculature. A potential advantage of using the Navien catheter over other devices for intracranial application lies in the navigability, especially for large thrombotic burdens with a high risk of thrombotic fragmentation. Nevertheless, the Navien microcatheter needs proximal support like a long guiding sheath.

In summary, CVST-related cerebral hemorrhage may occur in the early pregnancy period, with nonspecific clinical manifestations. The application of a 6 Fr Navien catheter and balloon angioplasty offers efficient effects for intraluminal recanalization of the thrombus-occluded cerebral venous sinuses, and continuous use of local urokinase through the microcatheter allows complete thrombolysis of residual wall-adherent clots. Endovascular recanalization with balloon angioplasty, aspiration, and local thrombolysis of thrombosed cerebral venous sinuses can be safely performed without the need of decompressive hemicraniectomy in early-pregnancy patients with malignant brain edema caused by progressive CVST.

## Author contributions

Study Design: Zi-Liang Wang

Data collection: Kun Zhang, Tian-Xiao Li

Data analysis: Kun Zhang, Bu-Lang Gao

Supervision: Liang-Fu Zhu

Validation: all authors

Approval: all authors

Wiring of the paper: Bu-Lang Gao
